# Kirk’s Hand-Book of Physiology

**Published:** 1889-06

**Authors:** 


KIRK'S HAND-BOOK OF PHYSIOLOGY, by Morrant Baker, F. R. C. S., Surgeon to St. Bartholomew's Hospital; Member of the Court of Examiners of the Royal College of Surgeons; Examiner in Surgery in the University of London and at the Royal College of Physicians; Late Lecturer on Physiology at St. Bartholomew's Hospital and Vincent Dormer Harris, M. D., London. Fellow of the Royal College of Physicians; examiner in Elementary Physiology at the Conjoint board of the Royal College of Physicians and Surgeons; Demonstrator of Physiology at St. Bartholomew's Hospital; Physician to the Victoria Park Hospital for diseases of the chest. Twelfth edition rearranged, revised and rewritten, and with five hundred illustrations.

New York, William Wood & Co., 1889.

The issue of a fresh edition of this excellent work on physiology, so soon after the preceding one, would seem to indicate not only a demand for the work, but a determination of the authors to keep fully abreast with the advances in the science, and to maintain the high standard which Kirk's Physiology has long deservedly held.

In the present volume there have been important alterations and changes made and considerable new matter, and about fifty new illustrations added. The work now contains 784 pages of clear, able and condensed matter, and while sufficiently full is yet free from useless detail, and presents that plainness of description and proper medium as to size, which the student needs. The order of subjects and the arrangement of the work we regard as admirable and without attempting here a detail of its merits we will simply say that, all things considered, we regard it as among the very best of all the text books on physiology now extant.

